# Genetic and Immunological Profiling of Recent SARS-CoV-2 Omicron Subvariants: Insights into Immune Evasion and Infectivity in Monoinfections and Coinfections

**DOI:** 10.3390/v17070918

**Published:** 2025-06-27

**Authors:** Nadine Alvarez, Irene Gonzalez-Jimenez, Risha Rasheed, Kira Goldgirsh, Steven Park, David S. Perlin

**Affiliations:** Center for Discovery and Innovation, Hackensack Meridian Health, 111 Ideation Way, Nutley, NJ 07110, USA; irene.gojim@gmail.com (I.G.-J.); risha.rasheed@hmh-cdi.org (R.R.); kira.goldgirsh@hmh-cdi.org (K.G.); steven.park@hmh-cdi.org (S.P.)

**Keywords:** SARS-CoV-2, Omicron, respiratory viruses, neutralization, human bronchial airway epithelial cells, ALI model

## Abstract

The evolution of the severe acute respiratory syndrome coronavirus 2 (SARS-CoV-2) and its impact on public health continue to demand attention as the virus continues to evolve, demonstrating a remarkable ability to adapt to diverse selective pressures including immune responses, therapeutic treatments, and prophylactic interventions. The SARS-CoV-2 variant landscape remains dynamic, with new subvariants continuously emerging, many harboring spike protein mutations linked to immune evasion. In this study, we characterized a panel of live SARS-CoV-2 strains, including those key subvariants implicated in recent waves of infection. Our findings revealed a significant variability in mutation patterns in the spike protein across the strains analyzed. Commercial antibodies and human convalescent plasma (HCoP) samples from unvaccinated donors were ineffective in neutralizing the most recent Omicron subvariants, particularly after the emergence of JN.1 subvariant. Using human airway epithelial cells derived from healthy bronchiolar tissue (hBAEC), we established both monoinfections and coinfections involving SARS-CoV-2, Influenza A virus H1N1 (IFAV_H1N1) and Respiratory Syncytial Virus (RSV). Assessments were conducted to compare viral infectivity and the production and release of immune mediators in the apical and basolateral compartments. Notably, Omicron KP.3.1.1 subvariant induced a more pronounced cytopathic effect in hBAEC compared to its parental strain JN.1 and even surpassed the impact observed with the ancestral wild-type virus (WA1/2020, Washington strain). Furthermore, the coinfection of KP.3.1.1 subvariant with IFAV_H1N1 or RSV did not attenuate SARS-CoV-2 infectivity; instead, it significantly exacerbated the pathogenic synergy in the lung epithelium. Our study demonstrated that pro-inflammatory cytokines IL-6, IFN-β, and IL-10 were upregulated in hBAEC following SARS-CoV-2 monoinfection with recent Omicron subvariants as well as during coinfection with IFAV_H1N1 and RSV. Taken together, our findings offer new insights into the immune evasion strategies and pathogenic potential of evolving SARS-CoV-2 Omicron subvariants, as well as their interactions with other respiratory viruses, carrying important implications for therapeutic development and public health preparedness.

## 1. Introduction

Five years after Coronavirus Infectious Disease 2019 (COVID-19) was declared a pandemic by the World Health Organization (WHO), SARS-CoV-2 remains as one of the most prevalent respiratory infections worldwide, with greater morbidity and mortality than other respiratory viruses that have been circulating for decades [1].

During the early phase of the pandemic, convalescent plasma therapy was successfully administered, particularly to patients who responded poorly to antiviral treatments. However, the emergence of new subvariants has increasingly undermined the effectiveness of antibody-based therapies, reducing their ability to prevent severe disease outcomes [2].

**Figure 1:**
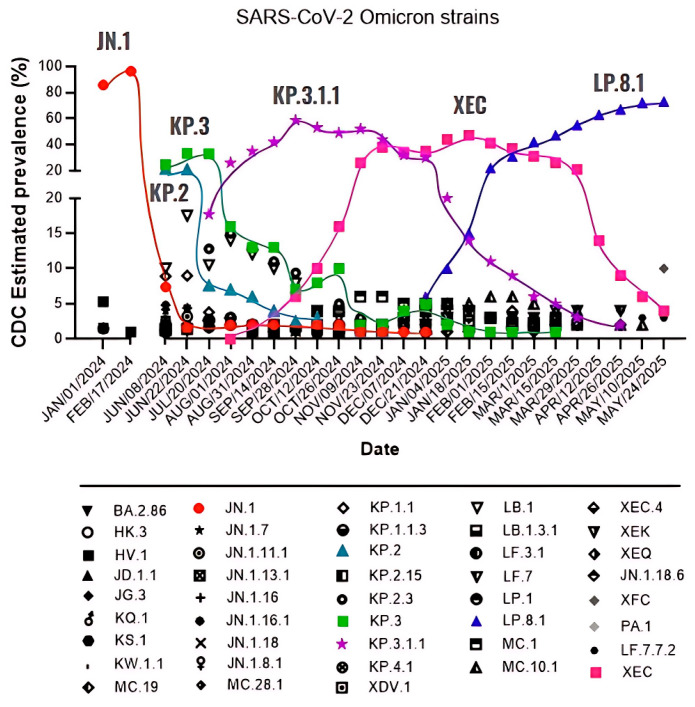
The SARS-CoV-2 Omicron variant is continuously evolving. From January 2024 to March 2025, the Omicron variant was responsible for the different waves of prevalence, which are represented here by JN.1, KP.2, KP.3, KP.3.1.1, XEC, and LP.8.1. Information extracted from the Centers for Disease Control (CDC) bi-weekly update [6].

Despite the current interventions for SARS-CoV-2 infection including vaccines, antiviral drugs, monoclonal antibodies, corticosteroids, and other pharmacological agents, the high transmissibility of SARS-CoV-2 drives continuous evolution of its genome, with new mutations emerging, particularly in immune-relevant regions such as spike protein [3,4]. Specifically in the spike protein, the receptor-binding domain (RBD) and the N-terminal domain (NTD) are key regions where mutations highly affect the effectiveness of neutralizing antibodies [5]. Since the onset of the pandemic, SARS-CoV-2 has rapidly evolved into multiple variants, with Omicron serving as the parent lineage for nearly all currently circulating subvariants. A comprehensive view of the evolutionary landscape of SARS-CoV-2 since the emergence of Omicron JN.1 is shown in Figure 1.

Besides this, SARS-CoV-2 has been circulating in association with other respiratory viruses, but the prevalence of these coinfections has been underestimated due to the global focus being primarily directed toward SARS-CoV-2. Currently, SARS-CoV-2 coinfections with IFAV_H1N1 and RSV are increasingly recognized [7,8,9,10,11], but further studies are needed to better understand the extent of lung tissue damage caused by these coinfections and their implications for host immunity.

Here, we monitored the prevalence of emerging SARS-CoV-2 Omicron subvariants across the United States to identify key spike mutations in strains driving the recent waves of infection and evaluate their infection phenotypes. We assessed the efficacy of commercially available monoclonal antibodies and HCoP samples against a panel of recently emerged SARS-CoV-2 strains, including clinical isolates obtained within New Jersey (NJ). Several recent studies have evaluated SARS-CoV-2 infection in human airway epithelial cells (hAECs), both as a monoinfection and in combination with other respiratory viruses [12,13,14,15,16]. However, there is currently no available characterization of infections caused by the most recent Omicron subvariants, nor are there reports on coinfections involving these strains and other respiratory viruses in human bronchial cells.

To the best of our knowledge, this is the first study to evaluate the effects of viral infection by the recent Omicron subvariants JN.1 and KP.3.1.1 in the bronchial region of the respiratory system using hBAEC. It is also the first report to investigate the coinfection of Omicron KP.3.1.1 with IFAV_H1N1 and RSV, using hBAEC in an air–liquid interface (ALI) model. Our findings offer new insights into the pathogenicity and immune evasion mechanisms of recent Omicron subvariants and their interactions with other respiratory viruses, with important implications for public health preparedness and the development of therapeutic strategies.

## 2. Materials and Methods

### 2.1. Cell Lines

African green monkey kidney cells (VeroE6/TMPRSS2) were obtained from XenoTech, Japan (Cat. No. JCRB1819, Lot No. 2222020). Madin-Darby Canine Kidney (MDCK) cells were purchased from the Biodefense and Emerging Infections Research Resources repository (BEI Resources, Manassas, VA, USA, Cat. No. NR-2628, Lot No. 494646-2). Both VeroE6/TMPRSS2 and MDCK cells were maintained in high-glucose Dulbecco’s Modified Eagle Medium (DMEM, ATCC Cat. No. 30-2002TM), supplemented with 10% fetal bovine serum (FBS) (Thomas Scientific, Swedesboro, NJ, USA, Cat. No. C788U22) and 1% antibiotic/antimycotic (A/A) (ThermoFisher, Waltham, MA, USA, Cat. No. 15240062). Human cervix epithelial cells (Hep-2) were purchased from Milipore Sigma (Rockville, MD, USA, Cat. No. 86030501-1VL, Lot. No. 16K049) and maintained in Eagle’s Minimum Essential Medium (EMEM, ATCC Cat. No. 30-2003TM, ATCC, Manassas, VA, USA), supplemented with 10% FBS and 1% A/A. All cell lines were cultured as monolayers at 37 °C with 5% CO_2_ and appropriate humidity and sub-cultured at regular intervals to maintain their exponential growth using recommended split ratios and medium replenishment volumes.

### 2.2. Viral Infections, Cytopathic Effect and Stock Preparation

#### 2.2.1. SARS-CoV-2

SARS-CoV-2 virus strains were obtained from BEI Resources and through the Hackensack Meridian Health BioRepository (HMH-BioR). All HMH-BioR strains were recovered from nasopharyngeal swabs collected by the NJ Department of Health (NJDOH) surveillance program for COVID-19 and were confirmed as SARS-CoV-2 by whole-genome sequencing performed at the New York Genome Center. In this study, the WA1/2020 strain was used as the reference for SARS-CoV-2, as it was isolated from the oropharyngeal swab of a patient who returned from an affected region in China and later developed clinical disease (COVID-19) on 19 January 2020, in Washington, DC, USA. The source of each individual strain is listed in Table S1. For virus propagation, 2 × 10^6^ VeroE6/TMPRSS2 cells were seeded in a T25 flask one day prior to infection under the conditions described previously. Once the cells formed a confluent monolayer, the media were removed, and 100 µL of the virus inoculum was added to the flask allowing the infection for 1 h at 37 °C with 5% CO_2_. After incubation, the flask was replenished with fresh and warmed DMEM supplemented with 10% FBS and 1% A/A.

#### 2.2.2. Influenza A H1N1

Influenza A virus-A/WSN/33 (H1N1) PA-2A-Nluc (PASTN) (abbreviated here as IFAV_H1N1) was obtained from BEI Resources (Cat. No. NR-49383, Lot. No. 70037384). For virus propagation, 1.5 × 10^6^ MDCK cells were seeded in a T25 flask with DMEM supplemented with 10% FBS and 1% A/A. After 24 h, the cell growth medium was removed, and the cells were washed twice with virus propagation medium EMEM supplemented with 0.125% bovine serum albumin (BSA, ThermoFisher, Cat. No. 15260037) and 2 µg/mL TPCK-treated trypsin from bovine pancreas (TPCK-treated trypsin, Sigma, Cat. No. T1426). The cells were then infected with 100 µL of the virus inoculum and incubated for 1 h at 37 °C with 5% CO_2_. Following the incubation, the flask was replenished with the virus propagation medium.

#### 2.2.3. Respiratory Syncytial Virus

Recombinant Respiratory Syncytial Virus (RSV) A2 Expressing Green Fluorescent Protein rgRSV224 (abbreviated here as RSV) was obtained from BEI Resources (Cat. No. NR-52018, Lot. No. 70059814). For virus propagation, Hep2 cells were cultured in a T25 flask using an EMEM medium supplemented with 10% FBS and 1% A/A. Infection was carried out with 100 µL of the virus inoculum in 2 mL of EMEM supplemented with 2% FBS and 1% A/A, followed by 2 h incubation at 37 °C with 5% CO_2_. After the incubation, the flask was replenished with EMEM supplemented with 10% FBS and 1% A/A.

All viruses were incubated at 37 °C with 5% CO_2_. The cytopathic effect (CPE) was monitored daily at 4× magnification using a phase contrast microscope (EVOS XL Core Imaging System, Cat. No. AMEX1000, Thermo Fisher Scientific, Bothell, WA, USA). Virus stocks were prepared by collecting the supernatants when 70–80% CPE was observed. The cell debris was clarified by centrifugation at 1500 rpm for 5 min, and 500 µL of the supernatants were transferred into screw-cap tubes and stored at −80 °C until further use.

### 2.3. Plaque Forming Unit Assay (PFU)

Virus titration was performed based on a method previously published [17], with some modifications. For SARS-CoV-2, VeroE6/TMPRSS2 cells were seeded in 24-well plates at a density of 2.5 × 10^5^ cells in 500 µL per well and incubated overnight at 37 °C with 5% CO_2_. Ten-fold serial dilutions of the virus stock were prepared in DMEM with 10% FBS and 1% A/A. The cell monolayer was rinsed with the same medium, and 100 µL of the virus dilutions were added to the corresponding wells. The infection was allowed for 1 h at 37 °C with 5% CO_2_, with gentle rocking of the plates every 15 min. After 1 h, 500 µL of a pre-warmed overlay mixture (2× MEM with 2.5% Cellulose, 1:1) were added per well, and plates were incubated at 37 °C with 5% CO_2_ for 72 h. Finally, plates were fixed with 500 µL of 10% neutral buffered formalin (NBF, Thermo Fisher Scientific, Bothell, WA, USA) for 24 h and stained with 0.5% crystal violet (CV).

For IFAV_H1N1, MDCK cells in DMEM with 10% FBS and 1% A/A were seeded in a 24-well plate at the concentration of 2.5 × 10^5^ cells/well, followed by overnight incubation at 37 °C with 5% CO_2_. Viral dilutions were prepared in EMEM with 0.125% BSA and 2 ug/mL TPCK-treated trypsin. Cells were inoculated with 100 µL of each dilution, incubated for 1 h at 37 °C with 5% CO_2_, followed by the addition of overlay media containing 2% low melting point agarose (Lonza, Basel, Switzerland, Cat. No. 50100) in virus propagation medium (1:1). Finally, cells were fixed with 10% NBF at 4 days post-infection during 24 h and stained in 0.5% CV.

For RSV, 2.5 × 10^5^ Hep2 cells in EMEM with 10% FBS and 1% A/A were used for seeding and viral dilutions were prepared in EMEM with 2% FBS and 1% A/A. The incubation with the virus was extended to 2 h followed by an addition of an overlay containing 0.3% agarose in EMEM with 10% FBS and 1% A/A. Plates were then incubated for 6 days, and the staining was performed using 0.05% neutral red for 1 h. For all viruses, viral plaques were counted visually, and the virus titer was determined as PFU/mL.

### 2.4. Antibodies and Human Convalescent Plasma

Three commercially available antibodies were purchased from different sources: (1) Anti-SARS-CoV-2 Spike Receptor Binding Domain Broadly Neutralizing Antibody, Human IgG3 (AM359b) (Acro Biosystems, Newark, DE, USA, Cat. No. SPD-M400a); (2) Casirivimab Recombinant Human Monoclonal Antibody, SARS-CoV-2 RBD, IgG1 (ThermoFisher, Cat. No. MA5-54856); (3) Bebtelovimab Humanized Recombinant Human Monoclonal Antibody, SARS-CoV-2 RBD, IgG1 (ThermoFisher, Cat. No. MA5-54843). The antibody stocks concentrations were as follows: Anti-SARS-CoV-2 Spike RBD (600 µg/mL), casirivimab (1.7 mg/mL), and bebtelovimab (1.1 mg/mL). Additionally, HCoP samples (HUMC00007, HUMC00008, HUMC00009, and HUMC00011) were obtained from clinical specimens through the HMH-BioR, with 1000–10,000 IgG titers against the RBD of the SARS-CoV-2 spike protein. These samples were isolated from NJ patients that were infected with SARS-CoV-2 in 2020 and without receiving COVID-19 vaccination (HMH IRB. No. Pro2018-1022).

### 2.5. Neutralization Assay

Neutralization assays were performed as previously described [18], with minor adaptations. Briefly, VeroE6/TMPRSS2 cells were seeded 24 h prior to the assay in 96-well plates at a density of 1 × 10^4^ cells in 100 µL per well, followed by overnight incubation at 37 °C with 5% CO_2_. In a separate plate, two-fold serial dilutions of antibodies and HCoP samples were prepared in DMEM supplemented with 10% FBS and 1% A/A, to obtain the following final concentrations: 1, 0.5, 0.25, 0.125, and 0.062 µg/mL. An equal volume of SARS-CoV-2 virus was added to each dilution at a multiplicity of infection (MOI) of 0.1, and the antibody/plasma-virus mixtures were incubated for 1 h at 37 °C with 5% CO_2_. After this incubation, the media from the cell-containing plates were removed and replaced with 100 µL of the antibody/plasma-virus mixture, allowing interaction with the cells for 1 h at 37 °C with 5% CO_2_ and with gentle plate rotation every 15 min to ensure even distribution of the mixture. After this time, the antibody/plasma-virus mixture was removed, each well was replenished with 100 µL of fresh DMEM supplemented with 10% FBS and 1% A/A medium, and plates were incubated for 72 h following the same conditions. The CPE was monitored daily, and on day 3 post-infection, 100 µL of CellTiter-Glo 2.0 Cell Viability Assay (Promega, Madison, WI, USA, Cat. No. G9241) were added per well. The 50% neutralization titers (NT_50_) were determined from the IC_50_ values of each sample, normalized against control cells infected without antibody or plasma treatment. 

### 2.6. Spike Protein Target-Based Sequencing

RNA extraction was performed using a QiaCube HT instrument (QIAGEN, Hilden, Germany, Cat. No. 9001896, with QIAGEN 96-well format Cat. No. 9001896) and RNA extraction kit “QIAamp 96 Virus QIAcube HT Kit” (QIAGEN Cat. No. 57731), following the manufacturer’s instructions. Samples were lysed under highly denaturing conditions at room temperature (RT) in the presence of proteinase K and lysis buffer ACL, which together ensure the inactivation of nucleases. Buffer ACB was then added to adjust the binding conditions for RNA purification. The lysate was transferred to a QIAamp 96 plate (QIAGEN, Hilden, Germany), and nucleic acids were adsorbed onto the silica membranes under vacuum while contaminants passed through. Three wash steps effectively removed the remaining contaminants and enzyme inhibitors, and the RNA was eluted in buffer AVE. For cDNA synthesis, a PrimeScript RT Reagent Kit with gDNA Eraser (Takara, Cat. No. RR 047A) was used. The process was carried out in two steps: genomic DNA (gDNA) was eliminated using the gDNA Eraser at 42 °C, followed by reverse transcription with the PrimeScript RT Mix I at 37 °C.

To identify gene modifications in the spike protein of SARS-CoV-2, the full coding sequence of the open reading frame 2 (ORF2) gene was amplified and sequenced. PCR amplification was performed using the Takara^®^ PrimeSTAR HS Kit (Takara Bio Inc., San Jose, CA, USA, Cat. No. R010B) according to the manufacturer’s instructions, with the primers listed in Table S1. The PCR conditions were as follows: an initial denaturation cycle at 98 °C for 10 min, followed by 35 cycles consisting of 15 s of annealing at 54 °C, a 2 min and 10 s elongation at 72 °C, and a final extension at 72 °C for 10 min. PCR products were verified by 0.8% agarose gel electrophoresis and subsequently sent for Sanger sequencing to Genewiz^®^ (South Plainfield, NJ, USA) using the same primers. The resulting sequences were analyzed using Seqman Pro version 17.5.0 (Lasergene DNAStar^®^ software, Madison, WI, USA).

### 2.7. Ex Vivo Air–Liquid Interface Model (ALI)

#### 2.7.1. Human Bronchial Airway Epithelial Cells (hBAECs)

Sterile hBAEC were purchased from Epithelix (Geneva, Switzerland, Cat. No. EP51AB, Batch No. 02AB0940), after being isolated from a 62-year-old female donor, with non-smoking record and no reported pathology, and cryopreserved as passage 1 in October of 2022. Cells were cultivated in PneumaCult™ ExPlus Expansion Media (StemCell, Vancouver, BC, Canada, Cat. No. 05040), supplemented with 0.1% hydrocortisone (Stemcell Cat. No. 07925) and incubated at 37 °C with 5% CO_2_. After 3 days, the cells were washed with D-PBS (without Ca++ and Mg++) (Sigma Cat. No. D8537), and the detachment was achieved after using ACF Enzymatic Dissociation Solution for 7 min at 37 °C, followed by the addition of ACF Enzyme Inhibition Solution used to neutralize the reaction (StemCell Cat. No. 05426). The cell suspension was centrifuged for 5 min at 360× *g*, and the pellet was resuspended in PneumaCult™Ex Plus Medium. Cells were then expanded into 24-well plates using transwell collagen-coated inserts (Corning, Corning, NY, USA, Cat. No. 3495), with a polyester (PET) membrane of 0.4 uM pore size and a surface area of 0.33 cm^2^. Each insert was seeded with a density of 3.3 × 10^4^ cells in 200 µL of PneumaCult™-ExPlus Medium. Once 70–80% confluence was reached, the cells were airlifted, with replacement of the basolateral medium every 2–3 days using PneumaCult™-ALI Maintenance Medium supplemented with 0.2% heparin (Stemcell Cat. No. 07980) and 0.5% hydrocortisone (StemCell Cat. No. 07925). The differentiation of the epithelium was monitored using a phase contrast microscope (EVOS XL Core Imaging System, Cat. No. AMEX1000).

#### 2.7.2. Monoinfection and Coinfection Assays in Human Bronchial Airway Epithelial Cells

After 23–28 days of maturation, including mucus production and cilia beating, two independent hBAEC inserts were used to evaluate the effect of monoinfections (WA1/2020, JN.1 and KP.3.1.1) and coinfections (KP.31.1+IFAV_H1N1 and KP.31.1+ RSV). The apical surface of hBAEC was inoculated with 2 × 10^5^ PFU/mL per insert of each corresponding virus in 200 µL of PneumaCult™-ALI Basal Medium (STEMCELL Technologies Inc., Vancouver, BC, Canada). For coinfections, 2 × 10^5^ PFU/mL of each virus strain were mixed and 200 µL from the mixture was used for infection. Plates were incubated for 2 h at 35 °C with 5% CO_2_ to allow virus internalization. After incubation, the virus inoculum was collected and evaluated by PFU assay to determine the effectiveness of hBAEC infection. Additional apical washes were performed to eliminate the unbound virus, with the last wash being collected and used as the day-zero sample. Subsequently, plates were returned to the incubator at 37 °C with 5% CO_2_, and CPE was monitored every 24 h, with collection of apical and basolateral washed in each time point. Mock cells were incubated with the growth culture medium during the infection step. All apical and basolateral samples from each time point were stored at −80 °C, and ALI cultured cells were fixed with 4% formaldehyde (*w*/*v*) (ThermoFisher Cat. No. J61899.AP) in both apical and basolateral chambers and conserved at 4 °C until further processing.

#### 2.7.3. Viral Quantification by Quantitative Reverse Transcription-PCR (RT-qPCR)

Apical and basolateral samples were inactivated with proteinase K (1:10 ratio) for 1 h at 65 °C. Viral RNA was isolated from the samples, using the Qiagen QIAcube HT (Germantow, MD, USA) automated mid-to-high-throughput nucleic acid purification instrument with the QIAamp 96 Virus QIAcube HT Kit (Cat. No. 57731). For SARS-CoV-2, RT-qPCR was performed using the E gene primer, probe panel, and RNase P gene as described before [19]. For IFAV_H1N1, primers and probes or iTaq Universal SYBR Green One-Step Kit (Bio-Rad, Hercules, CA, USA, Cat. No. 172-5151) targeting the haemagglutinin (HA), neuraminidase (NA) and matrix (M) genes were used, using the sequences included in the WHO report of 2021 [20]. For RSV, F and N proteins were amplified using primers and probes described in the literature [21]. All the sequences related to the three viruses are listed in Table S2. The limit of quantification (LOQ) was calculated using the standard error (SE) and standard deviation (SD) of the intercept from the standard curves of the RT-qPCR, and it was determined as 1 log of quantity of copies for this assay.

#### 2.7.4. hBAEC Differentiation and Colocalization Evaluated by Immunofluorescence Assay

The differentiation of the hBAEC epithelium was assessed using fluorescence microscopy and specific differentiation markers. Three of 1X PBS washes were applied to the fixed cells, followed by blocking and permeabilization steps with 3% BSA (Sigma Aldrich, Saint Louis, MO, USA, Cat. No. A7906) and 0.03% Triton X-100 (Sigma Aldrich Cat. No. X100-5ML) for 30 min at RT. Immunofluorescence staining was performed for the following cell markers diluted in blocking buffer: tight junction protein, ZO1 (1:100 dilution, mouse monoclonal, Alexa Fluor 555 conjugate; ThermoFisher, Cat. No. MA3-39100-A555), and goblet cell marker, Muc5ac (1:100 dilution, mouse monoclonal, Alexa Fluor^®^ 647 conjugate; Abcam, Cambridge, MA, USA, Cat. No. ab309611). After the addition of each antibody conjugate, cells were incubated at RT with gentle agitation and washed three times using 0.1% Triton X-100 in 1X PBS. For the SARS-CoV-2 nucleocapsid staining, blocked and permeabilized cells were incubated with the primary antibody (anti-SARS-CoV-2 nucleocapsid antibody, 1:10,000 dilution; BioLegend, San Diego, CA, USA, Cat. No. A20087F) diluted in 2% BSA + 0.1% Triton X-100 in 1X PBS, incubated at 4 °C overnight with gentle agitation and followed by three washes with 0.1% Triton X-100 in 1X PBS. The secondary antibody conjugate (goat polyclonal, Alexa Fluor™ 488, ThermoFisher, Cat. No. A-11001) was added at 1:20,000, diluted in 5% BSA + 0.1% Triton X-100 in 1X PBS for 1 h at RT. Cells were finally washed three times with 0.1% Triton X-100 in 1X PBS, counter-stained with DAPI (4′,6-diamidino-2-phenylindole; ThermoFisher Cat. No. 62249), and visualized with acquisition of images at 20×, using Nikon Ti2 Epi-fluorescence microscope (Nikon Instruments Inc., Melville, NY, USA) and NIS Elements imaging software (Version 5.30.06).

#### 2.7.5. Profile of Cytokine Expression in hBAEC

To assess the cytokine expression profile in hBAEC, an RT-qPCR-based method was used for the detection of IL-6, TNF-α, IFN-β, and IL-10, including β-actin as a housekeeping reference gene for normalization. Apical and basolateral samples collected from all-time points were heat-inactivated at 56 °C for 1 h. The RT-qPCR was performed using the iTaq Universal SYBR Green One-Step Kit (Bio-Rad Cat. No. 172-5151) following manufacturer’s instructions. The primers sequences to target the cytokines were obtained from a previous study [22] and are listed in Table S2. The reverse transcription step was carried out at 50 °C for 15 min, followed by 40 cycles of PCR amplification on the AriaMx Real-Time PCR System (Agilent, Santa Clara, CA, USA, Cat. No. G8830A). The amplification conditions included denaturation at 95 °C for 15 s, annealing and extension at 52 °C for 30 s, followed by Melt Curve analysis.

### 2.8. Statistical Analysis

Statistical analyses were performed, and plots were generated using GraphPad Prism version 10.0.0 for Windows, GraphPad Software (Boston, MA, USA). For *p* value, *p* < 0.05 was considered significant for all statistical analyses. The viral particles obtained from the RT-qPCR assay were evaluated by comparing all time points from day 0 to day 4 (for SARS-CoV-2 and IFAV_H1N1) and to day 6 (for RSV), using ordinary one-way ANOVA followed by Dunnett’s multiple comparisons test. NT_50_ titers were calculated using non-linear regression analysis. To quantify the colocalization of Muc5ac and ZO-1 across the hBAEC imaged by widefield deconvolution immunofluorescence microscopy, we applied standard colocalization analysis, including Pearson’s correlation coefficient (PCC) and Mander’s colocalization coefficient (MCC) analyses [23], with statistical significance assessed using ANOVA followed by Tukey’s multiple comparisons test. To evaluate the cytokines expression compared to the housekeeping gene β-actin, the relative expression was calculated following the 2^−ΔΔCt^ Livak method [24], and the data were analyzed using ordinary one-way ANOVA followed by Tukey’s multiple comparison test.

## 3. Results

### 3.1. In Vitro Characterization of SARS-CoV-2 Infection Using VeroE6/TMPRSS2

A comprehensive collection of SARS-CoV-2 clinical isolates collected from more than 50,000 patients in NJ from 2020 to 2025, as part of an active regional surveillance program [25], has been curated at the Center for Discovery and Innovation (CDI). Additional isolates were obtained from BEI Resources. This panel includes the original WA1/2020 strain and all major SARS-CoV-2 variants and subvariants up to the time of this study. All virus strains were propagated in VeroE6/TMPRSS2 cells, where a strong CPE was visible by microscopy as early as four days post-infection, mainly characterized by disruption of the cell monolayer, cell rounding, and detachment. For the purposes of this study, only the infection with WA1/2020, JN.1, and its most prevalent descendants are included in Figure S1. Interestingly, a distinctive CPE was observed in JN.1 and its descendants (LB.1, KP.2.3, KP.3.1.1, and XDK.1) compared to the wild-type WA1/2020 strain, marked by a greater accumulation of dead cell aggregates, appearing as dark regions under the microscope (Figure S1a). Following each infection, viral stocks were collected and quantified using plaque assays with viral titers expressed as plaque-forming units per milliliter (PFU/mL) (Figure S1b). For coinfection experiments, Influenza H1N1 and RSV were also evaluated, with their respective CPE and viral titers shown in Figure S2a and S2b, respectively.

### 3.2. SARS-CoV-2 Omicron Subvariants Escape the Neutralizing Effects of Antibodies

**Figure 2:**
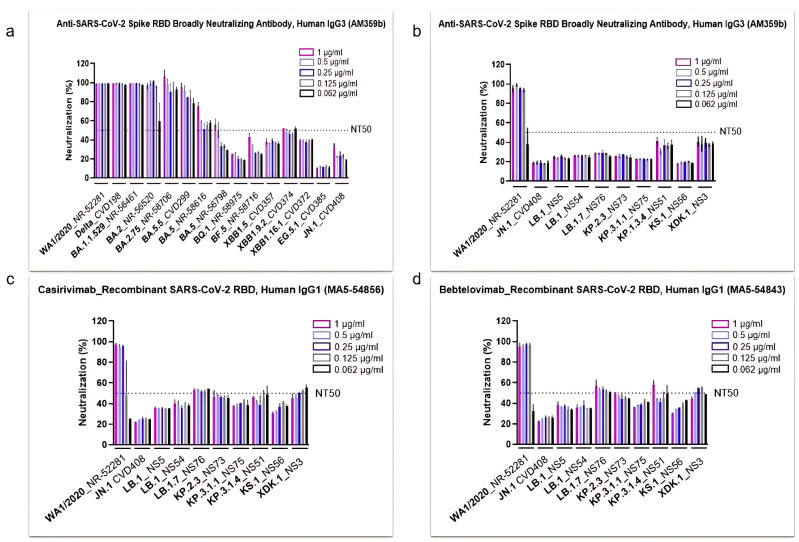
Commercial antibodies failed to efficiently neutralize the most recent SARS-CoV-2 Omicron subvariants. The neutralization efficacy of human antibody AM359b (anti-spike RBD) was assessed against SARS-CoV-2 subvariants circulating from the onset of the pandemic up until JN.1 (**a**), as well as against WA1/2020 and selected Omicron subvariants (**b**). Two recombinant SARS-CoV-2 Abs, casirivimab (**c**) and bebtelovimab (**d**), were evaluated against WA1/2020 and selected Omicron subvariants. All antibodies were tested with a concentration range from 1 to 0.062 µg/mL. NT_50_ represents the concentration required for a 50% neutralization activity.

To investigate the impact of different SARS-CoV-2 Omicron subvariants on immune evasion, the in vitro neutralization activity of several commercial antibodies was assessed (Figure 2). The anti-SARS-CoV-2 spike RBD antibody AM359b was first tested against major strains spanning from the original WA1/2020 isolate to the JN.1 subvariant (Figure 2a). Results showed that AM359b maintained strong neutralizing activity against WA1/2020 and early variants, including Delta through Omicron BA.2.75. However, starting with Omicron BA.5, the antibody’s neutralization capacity declined significantly, falling below 50% for emerging strains through JN.1 and dropping to less than 20% against EG.5.1, a derivative of XBB.1.9.2 (Figure 2a).

After observing this trend, we further profiled JN.1 and its descendants, using the AM369b Ab along with two clinically used antibodies (Abs), casirivimab and bebtelovimab (Figure 2b, Figure 2c, and Figure 2d, respectively). All three antibodies demonstrated strong neutralization activity against the parental (ancestral) WA1/2020 strain. However, the neutralization levels remained below 50% against nearly all the other evolved strains, with only a few exceptions marginally surpassing this threshold (Figure 2b–d). Interestingly, modest increases in neutralization were observed by some of the most recently emerged subvariants. Specifically, all three antibodies showed more activity against KP.3.1.4 and XDK.1, while casirivimab and bebtelovimab also demonstrated better neutralization against LB.1.7 and KP.2.3 (Figure 2c,d).

**Figure 3:**
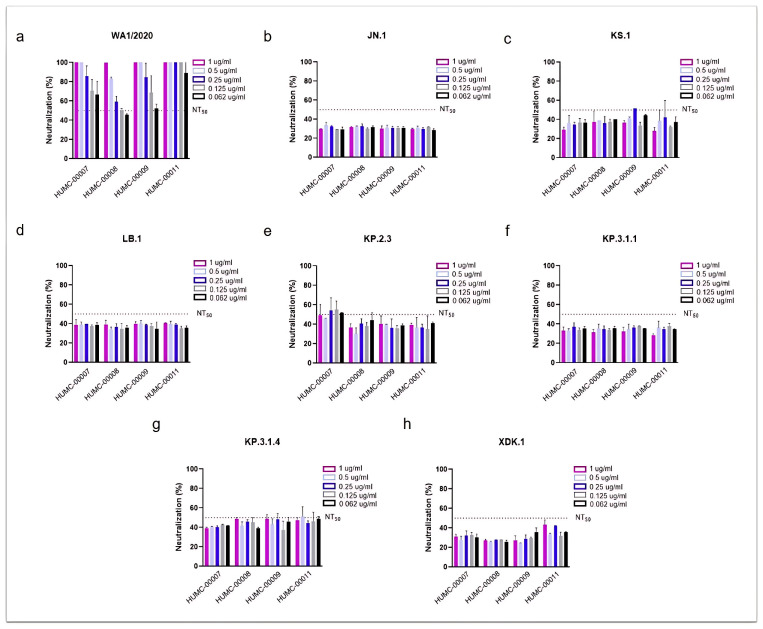
Human convalescent plasma (HCoP) from previous and unvaccinated donors does not provide protection against the newly emerging SARS-CoV-2 Omicron subvariants. The strains include SARS-CoV-2 WA1/2020, used as a reference (**a**), Omicron JN.1 (**b**), and the newly emergent subvariants (**c**–**h**). HUMC-00007, HUMC-00008, HUMC-00009, and HUMC-00011 (HCoP samples collected in 2020) were tested with a concentration range from 1 to 0.062 µg/mL. NT_50_ represents the concentration required for a 50% neutralization activity.

To assess whether human plasma from previously infected individuals exhibit neutralizing activity against newly circulating subvariants, we evaluated four HCoP samples with high IgG titers against the SARS-CoV-2 spike RBD (Figure 3). These samples were collected from NJ patients infected during the initial wave of the pandemic in 2020, prior to the availability of the COVID-19 vaccines.

As expected, all HCoP samples showed strong neutralizing activity against the ancestral WA1/2020 strain (Figure 3a), which was dominant at the time of samples collection. The NT_50_ values were achieved at the lowest concentration tested (0.062 µg/mL), with two samples (HUMC-00007 and HUMC-00011) demonstrating 60% and 90% neutralization at this minimal dose, respectively. In contrast, little to no neutralizing effect was observed against Omicron JN.1 and its descendants (KS.1, LB.1, KP.2.3, KP.3.1.1, KP.3.1.4, and XDK.1) (Figure 3b–h). In only a few instances the antibody showed 50% neutralization, such as HUMC-00007 against KP.2.3 (Figure 3e), and HUMC-00008, HUMC-00009, and HUMC-00011 against KP.3.1.4 (Figure 3g).

### 3.3. New Mutations, Rather than an Increase in the Number, Are Present in the Newly Emergent SARS-CoV-2 Subvariants

**Figure 4:**
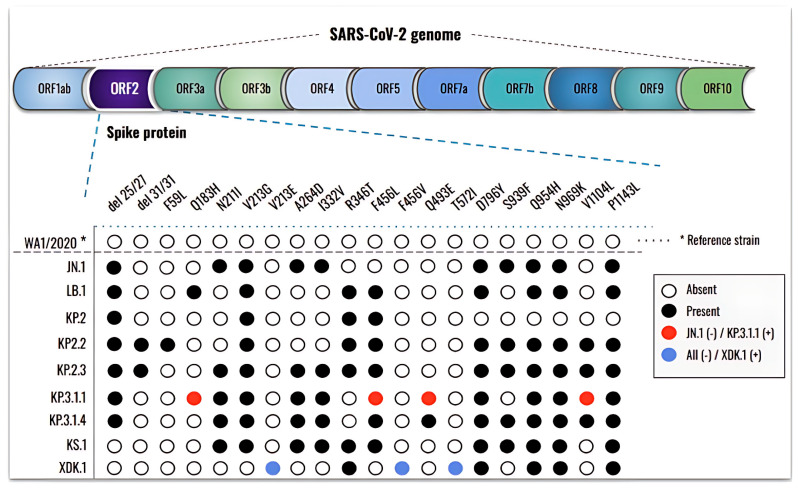
The new SARS-CoV-2 Omicron subvariants exhibit additional amino acid substitutions in the spike protein. The WA1/2020 strain (hCoV-19/USA-WA1/2020 BEI NR-52281) was retrieved from the GISAID database and used as a reference (*). The absence or presence of mutations in the spike protein ORF2 region is indicated by empty or black circles, respectively, while red circles indicate new mutations in KP.3.1.1 compared to JN.1 and blue circles indicate new mutations present in XDK.1.

To determine whether the differences among newly emerged SARS-CoV-2 Omicron subvariants correlate with changes in the spike protein, we sequenced the ORF2 region of their genomes (Figure 4).

Downstream analysis revealed numerous mutations across all subvariants when compared to the reference WA1/2020 strain, with more recent subvariants exhibiting a greater number of changes. Relative to Omicron JN.1, ten novel mutations were identified in its descendants: del31/31, F59L, Q183H, V213E, R346T, F456L, F456V, Q493E, T572I, and V1104L. Among these, LB.1 exhibited three new mutations (Q183H, R346T, and F456L) while KP.2.2 carried del31/31 and F59L. At the time of this study, KP.3.1.1 was the most prevalent subvariant and was therefore selected for direct comparison with JN.1. KP.3.1.1 harbored four mutations absent in JN.1 (Q183H, F456L, Q493E, and V1104L), while S939F was present in JN.1 but not in KP.3.1.1. Following the emergence of KP.3.1.1, additional subvariants continued to arise. Notably, XDK.1 was distinguished by three unique mutations (V213E, F456V, and T572I) that were not present in JN.1 or any of its prior descendants.

### 3.4. Omicron SARS-CoV-2 Induces a Strong Cytopathic Effect in hBAEC During Monoinfection or Coinfection with IFAV H1N1 and RSV

**Figure 5:**
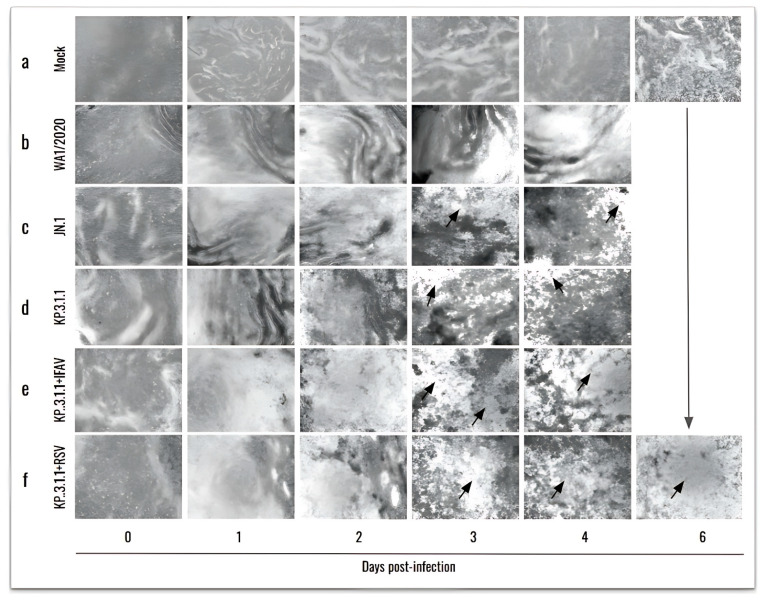
New SARS-CoV-2 Omicron subvariants induce high damage in the human bronchial airway epithelium, especially when co-inoculated with IFAV_H1N1 and RSV. From the top to the bottom, mock epithelium (uninfected), monoinfections (WA1/2020, Omicron JN.1, and Omicron KP.3.1.1), and coinfections (KP.3.1.1+IFAV_H1N1 and KP.3.1.1+RSV) were evaluated. From the left to the right, daily images were taken from 0 to 6 days. Cytopathic effects were identified by visual changes and disruption of the epithelial morphology, as indicated by the arrows. Images were captured at 4× magnification.

To investigate whether the recent SARS-CoV-2 Omicron subvariants JN.1 and KP.3.1.1 induce a significant infection in the human respiratory tract in comparison to the parent strain WA1/2020, we developed an ex vivo ALI model that mimics the lung epithelium using hBAEC from a bronchiolar source (Figure 5).

In parallel, considering the frequent co-circulation of multiple respiratory viruses in human populations, we also investigated the effects of SARS-CoV-2 coinfection with other clinically relevant pathogens, namely, Influenza A H1N1 and RSV. Cytopathic effect was evaluated in hBAEC beginning 1 h after infection (day 0; Figure 5a) and then monitored every 24 h during 4 days in the case of all monoinfections, and the coinfection with IFAV_H1N1 (Figure 5b, Figure 5c, Figure 5d and Figure 5e, respectively). However, the infection was extended to 6 days for the two inserts coinfected with KP.3.1.1 and RSV, since RSV requires a longer time to establish the infection (Figure 5f).

The efficiency of the infection was evaluated by PFU assay using the viral inoculum recovered from the inserts after 2 h of incubation (Figure S3). As observed, very small titers were obtained for all the mono- and coinfections, indicating that most of the viral particles were internalized in the hBAEC.

The microscopic analysis revealed that the infection with WA1/2020 induced mild CPE during the assay, which became more pronounced by day 3 and 4 (Figure 5b) in contrast to the uninfected control epithelium (Figure 5a). In comparison, both Omicron subvariants (JN.1 and KP.3.1.1) exhibited distinct CPE patterns during the same timeframe, characterized by localized epithelial disruptions and the formation of patch-like lesions (indicated by *black arrows* in Figure 5c and Figure 5d, respectively). More severe CPE was observed in the coinfection of KP.3.1.1 with IFAV_H1N1 and RSV, where large areas of the epithelial surface were severely damaged and disorganized, suggesting enhanced pathogenicity during coinfection (Figure 5e and Figure 5f, respectively).

### 3.5. Omicron Subvariants Induce a Higher Level of Infection in hBAEC Compared to the SARS-CoV-2 Parent Strain

**Figure 6:**
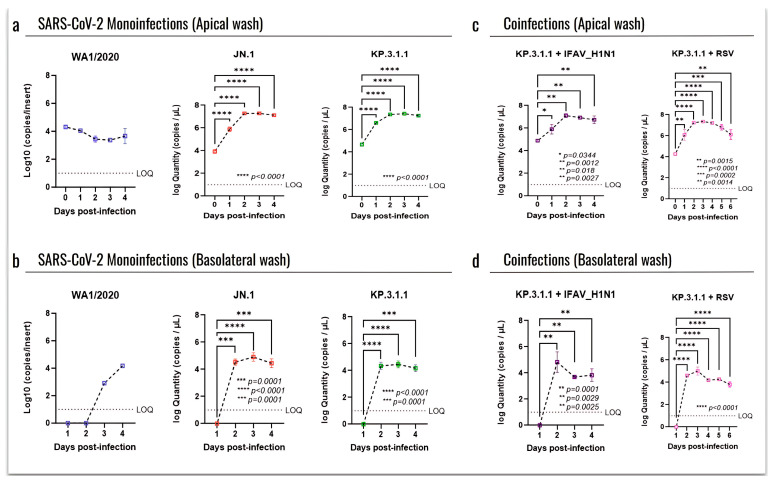
SARS-CoV-2 Omicron actively replicates in the human bronchial airway epithelium, both as monoinfection and in coinfection with IFAV_H1N1 and RSV. SARS-CoV-2 viral particles recovered from the hBAEC were quantified by RT-qPCR. Data are presented as the average of two technical replicates from two independent biological samples, with standard deviation (SD). (**a**) apical wash of hBAEC in WA1/2020, JN.1, and KP.3.1.1 monoinfections; (**b**) basolateral wash of hBAEC in WA1/2020, JN.1, and KP.3.1.1 monoinfections; (**c**) apical wash of hBAEC in KP.3.1.1+IFAV_H1N1 and KP.3.1.1+RSV coinfections; (**d**) basolateral wash of hBAEC in KP.3.1.1+IFAV_H1N1 and KP.3.1.1+RSV coinfections. SARS-CoV-2 viral RNA levels are expressed as copies per insert. LOQ indicates the limit of quantification for the RT-qPCR. The line above dots with an asterisk sign indicate a significant difference (*p* < 0.05) in the infection levels compared to day 0 in each assay.

To assess the infection of recent SARS-CoV-2 strains in the hBAEC, we compared the replication kinetics by RT-qPCR, related to an early circulating strain (WA1/2020). For this, the progression of infection was evaluated at each time point using day 0 as a baseline (Figure 6). Viral quantification from both apical and basolateral samples revealed very low infection induced by WA1/2020, with no progression in the apical side at day 0 and reaching only four logs in the basolateral side after 4 days of infection (Figure 6a and Figure 6b, respectively). In contrast, high levels of infection were detected in the apical samples after exposure to Omicron subvariants JN.1 and KP.3.1.1, with viral loads going from four to seven logs as early as 2 days after infection and with a plateau from day 2 to day 4 (*p* ≤ 0.0001) (Figure 6a). No significant differences were observed between the two Omicron subvariants in terms of infection recovered from the basolateral washes, where both peaked at day 2 with four logs and remained stable through the end of the assay (*p* ≤ 0.0001) (Figure 6b).

When evaluating the coinfections of KP.3.1.1 with the other common respiratory viruses, we observed that the combination with IFAV_H1N1 did not enhance the apical infection compared to the KP.3.1.1 monoinfection (Figure 6c). In fact, the coinfection with IFAV_H1N1 showed a peak on day 2, but the infection level dropped significantly by day 3 (*p* = 0.0029) and day 4 (*p* = 0.0025) (Figure 6d), different from the KP.3.1.1 monoinfection, which peaked on day 2 and remained stable through day 4 (*p* ≤ 0.0001) (Figure 6b). However, the combination with RSV showed a significant increase in infection through day 2 to day 4 compared to day 0 in the apical side (Figure 6c), and this significance was also evidenced in the basolateral compartment (Figure 6d).

**Figure 7:**
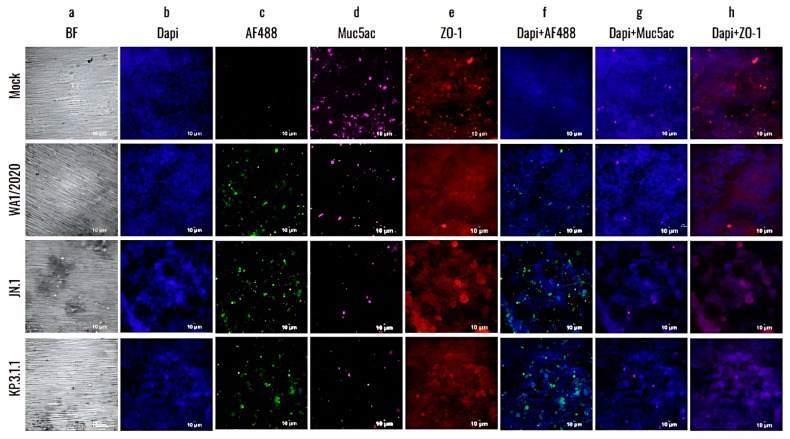
The newly emerged SARS-CoV-2 Omicron subvariants enhanced the infectivity and cytopathic effect in the human bronchial airway epithelium compared to the ancestral strain. hBAEC were infected with each corresponding SARS-CoV-2 strain at 2 × 10^5^ PFU/mL per insert. hBAEC infected with monoinfections (WA1/2020, JN.1 and KP.3.1.1) and coinfection with KP.3.1.1+IFAV_H1N1 were fixed on day 4 post-infection, while coinfection with KP.3.1.1+RSV was fixed on day 6 post-infection. Cells were stained with antibodies against cell markers to assess the epithelium differentiation (Muc5ac for goblet cells—*purple*- and ZO-1 for tight junctions—*red*); with anti-SARS-CoV-2 nucleocapsid antibody/goat polyclonal Alexa Fluor™ 488 (*green*) for viral antigen detection and counter-stained with the nuclear dye DAPI (*blue*). Images are visualized at 20× magnification and are representative of two independent inserts for each condition. The cross-section scale bar is 10 μm. BF, brightfield; AF, Alexa Fluor.

To further investigate the damage caused by SARS-CoV-2 monoinfections to the bronchial epithelium, the hBAEC were immuno-stained with differentiation markers that target goblet cells (Muc5ac) and tight junctions (ZO-1) and assessed the presence of infected cells using a specific anti-nucleocapsid antibody targeting SARS-CoV-2. As shown in Figure 7, the infection with Omicron JN.1 and KP.3.1.1 not only disrupted the epithelium (confirmed by BF observation and DAPI staining, Figure 7a,b) but also led to an increase in viral antigen detection when compared to WA1/2020 (Figure 7c). However, all infections induced a significant reduction in Muc5ac detection compared to the mock epithelium (Figure 7d). In addition, all the infected inserts showed a reduction in ZO-1 detection, but this effect was more pronounced in the case of JN.1 and KP.3.1.1, where instead of the regular intercellular connections, we observed cell aggregation and colocalization in multiple regions of the epithelium (Figure 7e). Merged images of staining with differentiation markers are provided to visualize the effects of all infections on hBAEC (Figure 7f–h).

**Figure 8:**
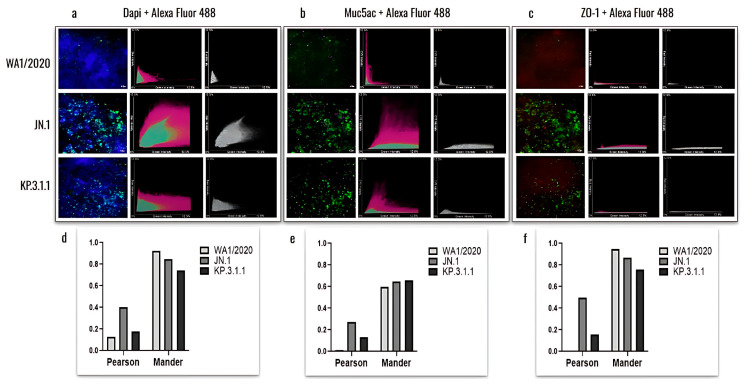
Colocalization analysis of hBAEC differentiation markers in SARS-CoV-2 infected tissues. Cells infected with WA1/2020, Omicron JN.1 and Omicron KP.3.1.1 were labeled with DAPI, Muc5ac and ZO-1 and co-stained with Alex Fluor 488. Merged and colocalization images are shown for each co-staining: (**a**) DAPI + Alexa Fluor 488; (**b**) Muc5ac + Alexa Fluor 488; (**c**) ZO-1+ Alexa Fluor 488. The scale bar for the merged images is 10 um and the intensity for the colocalization images is 12.5%. Pearson’s R values and the Mander’s overlap coefficients were obtained from the Nikon Ti2 Epi-fluorescence microscope and NIS Elements imaging software (Version 5.30.06). Analysis of variances (ANOVA) was performed followed by a Tukey’s multiple comparisons test with GraphPad Prism 10 (**d**–**f**).

Previous studies showed that goblet cells in human tracheobronchial epithelial cultures can be infected by the original SARS-CoV-2 Wuhan strain [13]. To assess whether hBAEC infection with Omicron subvariants exert any differential pattern compared to ancestral strain, we evaluated the colocalization of the SARS-CoV-2 nucleocapsid protein with Muc5ac (a goblet cell marker) and ZO-1 (a tight junction marker) (Figure 8).

The results showed a higher level of viral presence in hBAEC infected with Omicron subvariants JN.1 and KP.3.1.1 compared to those infected with the WA1/2020 strain, with Omicron JN.1 exhibiting the most prominent infection (Figure 8a). Scatterplots demonstrated that the viral nucleocapsid protein predominantly localized in Muc5ac-positive goblet cells, particularly in samples infected with Omicron JN.1 (Figure 8b). In contrast, immunofluorescence staining revealed minimal infection of ZO-1-positive cells by any of the SARS-CoV-2 subvariants (Figure 8c), suggesting that tight junctions are directly affected by WA1/2020 and the Omicron subvariants studied. The colocalization was analyzed by the Pearson correlation test and Mander coefficient’s overlap, but we did not find significant differences (Figure 8d–f).

### 3.6. Coinfection with Other Respiratory Viruses Does Not Reduce the Replication Capacity of SARS-CoV-2 in the Human Bronchial Airway Epithelium

**Figure 9:**
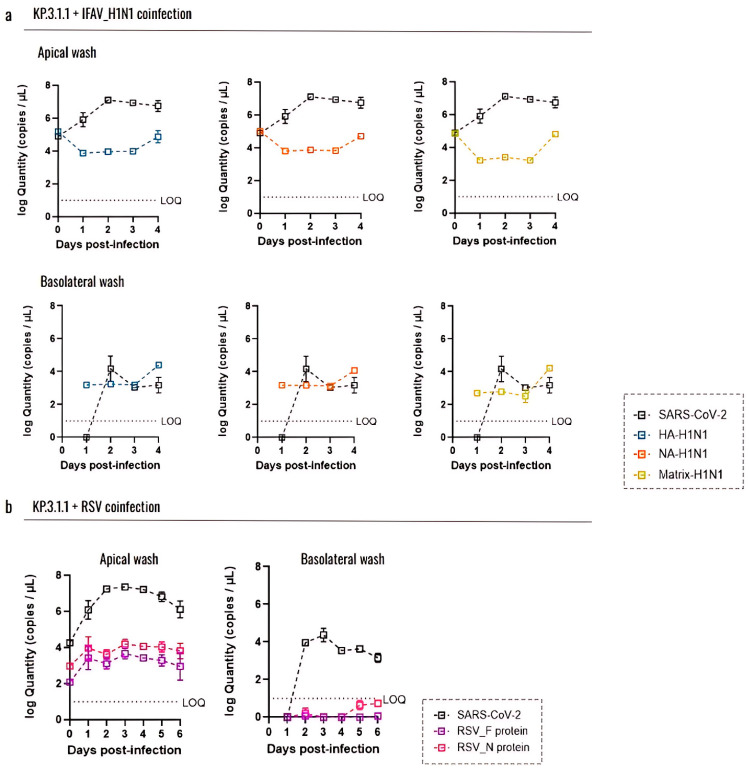
Coinfection with Influenza A H1N1 or RSV does not affect the replication capacity of SARS-CoV-2 Omicron in the hBAEC. Virus levels are expressed as log10 copies/mL. Graphs display the levels of (**a**) IFAV_H1N1 and KP.3.1.1 viral particles recovered from apical and basolateral wash samples, and (**b**) RSV and KP.3.1.1 viral particles recovered from apical and basolateral wash samples. Data are presented as the average of two technical replicates from two independent biological samples, with the standard deviation (SD). LOQ indicates the limit of quantification for the RT-qPCR.

To evaluate whether coinfection with IFAV_H1N1 or RSV impacts the SARS-CoV-2 replication, we quantified viral loads using RT-qPCR and compared it with SARS-CoV-2 viral RNA detection in the same inserts (Figure 9).

For IFAV_H1N1, three target genes (HA, NA, and matrix) were assessed (Figure 9a). In apical washes from inserts coinfected with KP.3.1.1 and IFAV_H1N1, viral levels followed similar trends across all three genes: a decrease from day 0 to day 1, a plateau through day 3, and a subsequent increase on day 4, with the matrix gene showing the most pronounced rise. However, these changes were not statistically significant. Notably, IFAV_H1N1 viral loads in apical washes were approximately half those of KP.3.1.1, indicating that SARS-CoV-2 remained the dominant virus in hBAEC cultures despite co-infection. In basolateral samples, IFAV_H1N1 was detectable from day 1, remained stable for 3 days, and increased on day 4, mirroring the apical trends (Figure 9a). For RSV, we targeted the F and N protein genes (Figure 9b). RSV levels in apical washes were low during coinfection with KP.3.1.1, with viral loads about half those of SARS-CoV-2. In the basolateral samples, only trace amounts of RSV were detected on days 5 and 6 post-infection, with values below the RT-qPCR assay’s limit of quantification (LOQ) (Figure 9b).

**Figure 10:**
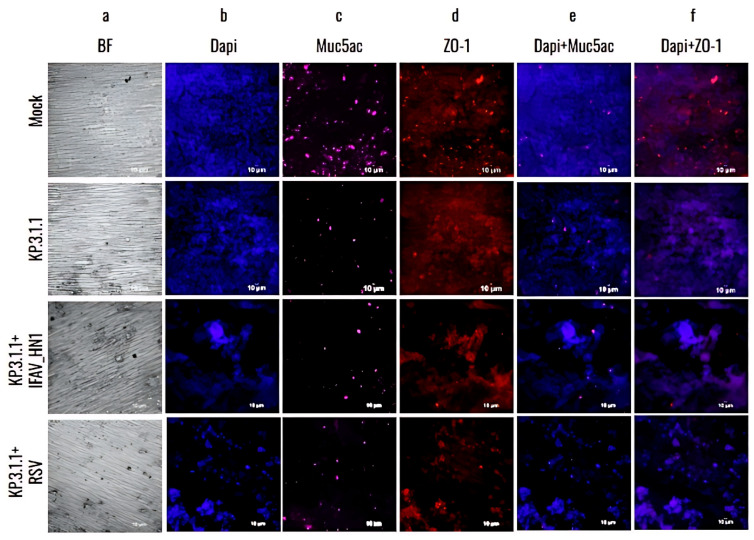
Coinfection of SARS-CoV-2 Omicron subvariant with Influenza A virus H1N1 or Respiratory Syncytial Virus affected the goblet cells and tight junctions in hBAEC. Cells were coinfected with KP.3.1.1+IFAV_H1N1 or KP.3.1.1+RSV at 2 × 10^5^ PFU/mL of each combination per insert (mock and KP.3.1.1 images are included as a side-by-side reference for the coinfections). Inserts were fixed on day 4 post-infection in the case of IFAV_H1N1 and on day 6 post-infection, in the case of RSV. hBAEC were stained with antibodies against cell markers to assess the epithelium differentiation (Muc5ac for goblet cells—*purple*- and ZO-1 for tight junctions—*red*), and counter-stained with the nuclear dye DAPI (*blue*). Images are visualized at 20× magnification and are representative of two independent inserts for each condition. The cross-section scale bar is 10 μm. BF, brightfield.

To validate the results obtained from RT-qPCR of coinfected inserts, hBAEC were additionally evaluated by immunofluorescence for the expression of epithelial differentiation markers Muc5ac and ZO-1 counterstained with DAPI (Figure 10). The CPE was more pronounced and damaging in KP.3.1.1+RSV in comparison to KP.3.1.1+IFAV_H1N1 (Figure 10a,b). The productive infection led to the reduction in Muc5ac detection and altered the expression of ZO-1, affecting the tight junctions and leading to cell aggregation suggesting possible disruption in the epithelial barrier integrity (Figure 10c,d). These observations were more evident in the case of coinfections of KP.3.1.1 with IFAV_H1N1 and RSV in comparison to monoinfection with KP.3.1.1, emphasizing that the coinfection of SARS-CoV-2 with the two other respiratory viruses aggravated the overall level of infection.

### 3.7. SARS-CoV-2 Mono- and Coinfection with IFAV_H1N1 and RSV Stimulate Strong Pro-Inflammatory Cytokine Response by hBAEC

Several types of host factors are stimulated during the progression of SARS-CoV-2 infection in humans, including inflammatory immune cells, cytokines and chemokines [26]. To investigate the immunological response of hBAEC upon SARS-CoV-2 mono- and coinfections, we measured the expression levels of selected proinflammatory cytokines based on their up- and down-regulation during SARS-CoV-2, IFAV_H1N1 and RSV infections [27,28,29].

**Figure 11:**
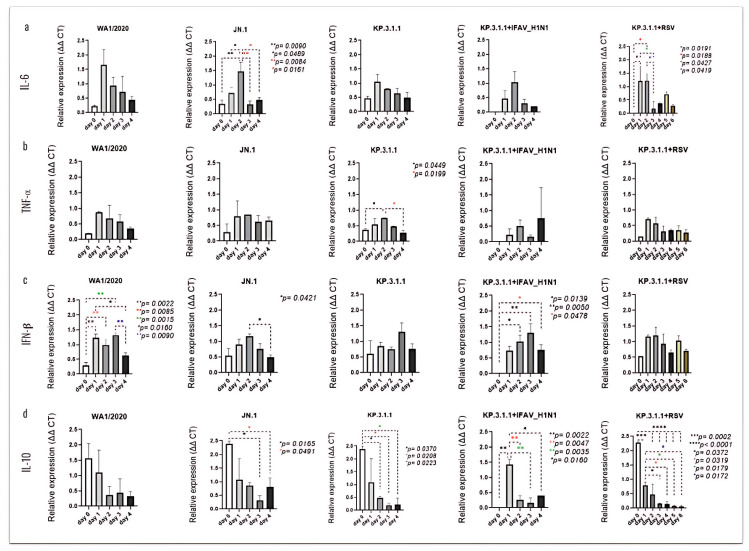
SARS-CoV-2 mono- and coinfections induce differential expression of immunological mediators in the apical side of the hBAEC. Apical washes were collected in each timepoint and analyzed for cytokine and chemokine secretion by PCR assay. (**a**) IL-6, (**b**) TNF*α*, (**c**) IFN-β, (**d**) IL-10. All measurements on y axis are represented as relative expression ΔΔCt. Data are presented as mean ± standard deviation (SD) of two independent inserts. Significant differences are indicated for *p* < 0.05 in the expression of the tested cytokines, when comparing all time points in each assay.

The analysis of differential cytokine expression following each mono- or coinfection revealed time-dependent changes, some of which were statistically significant (Figure 11). The inserts infected with JN.1 showed a peak of IL-6 on day 2 post-infection, with significance compared to day 0 (*p* = 0.0090), day 1 (*p* = 0.0489), day 3 (*p* = 0.0084), and day 4 (*p* = 0.0161) (Figure 11a). In the case of TNF-α, significant changes were observed only following hBAEC infection with KP.3.1.1, where levels peaked also at day 2 post-infection with *p* = 0.0449 vs. day 0 and *p* = 0.0199 vs. day 4 (Figure 11b). The infection with WA1/2020 led to a significant induction of IFN-β, with elevated expression as early as day 1 and a notable decrease by day 4 (Figure 11c). Specifically, levels on days 1 and 2 were significantly higher than day 0 (*p* = 0.0022 and *p* = 0.0085, respectively), while day 3 also differed from day 0 (*p* = 0.0015). Additionally, days 1 and 3 were significantly higher than day 4 (*p* = 0.0160 and *p* = 0.0090, respectively). High levels of IFN-β were also noticed after infection with JN.1, with a peak on day 2 and significant vs. day 4 (*p* = 0.0421). In the KP.3.1.1+IFAV_H1N1 coinfection, increasing levels of IFN-β were observed from day 1 to day 3. Expression on days 2 and 3 was significantly higher than on day 0 (*p* = 0.0139 and *p* = 0.0050, respectively). Although levels declined on day 4, they remained significantly elevated compared to day 0 (*p* = 0.0478) (Figure 11c). IL-10 was the most prominently induced cytokine on the apical side of hBAECs. The highest expression occurred on day 0 following infection with JN.1, KP.3.1.1, and the co-infection of KP.3.1.1+RSV, while peak expression was observed on day 1 for KP.3.1.1+IFAV_H1N1 (Figure 11d). All infections, except WA1/2020, showed a significant time-dependent decline in IL-10 levels by day 4.

**Figure 12:**
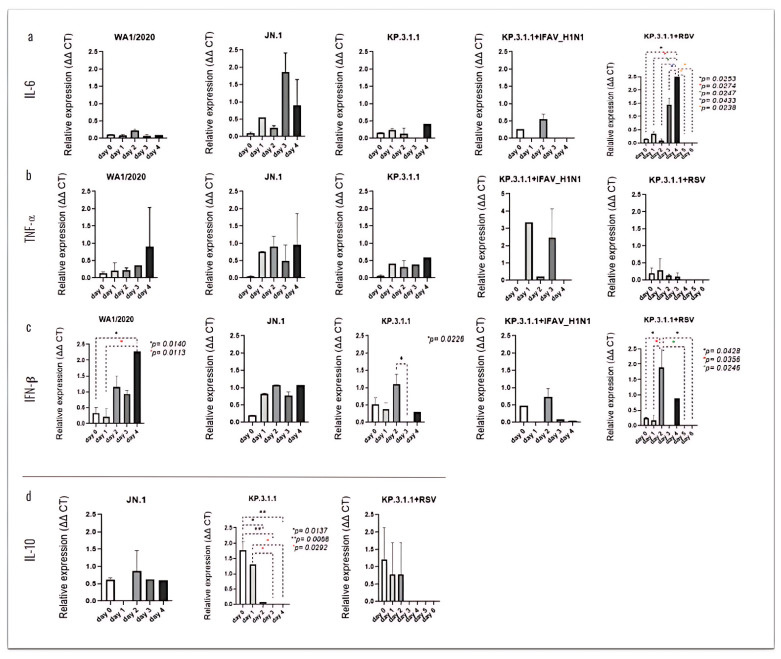
SARS-CoV-2 mono- and coinfections induce differential expression of immunological mediators in the basolateral side of the hBAEC. Basolateral media were collected in each timepoint and analyzed for cytokine and chemokine secretion by PCR assay. (**a**) IL-6, (**b**) TNF*α*, (**c**) IFN-β, (**d**) IL-10. All measurements on y axis are represented as relative expression ΔΔCt. Data are presented as mean ± standard deviation (SD) of two independent inserts. Significant differences are indicated for *p* < 0.05 in the expression of the tested cytokines, when comparing all time points in each assay.

To investigate the secretion of these cytokines through the hBAEC, we evaluated the release of these immunological mediators into the basolateral medium (Figure 12). Significant levels of IL-6 were observed after coinfection with KP.3.1.1+RSV, where the peak of expression was infected on day 4, showing significant levels compared to earlier and late time-points (Figure 12a). Similarly, infection with WA1/2020 induced the secretion of IFN-β in the basolateral side on day 4 post-infection, with *p* = 0.0140 vs. day 0 and *p* = 0.0113 vs. day 1 (Figure 12c). In the case of monoinfection with KP.3.1.1 or coinfection of KP.3.1.1+RSV, elevated levels of IFN-β were observed on day 2. For KP.3.1.1 monoinfection, expression on day 2 was significantly higher than on day 3 (*p* = 0.0226). The coinfection condition showed multiple significant differences compared to both earlier and later time points (Figure 12c).

The expression of IL-10 was higher on day 0 with significance against day 2 (*p* = 0.0137), day 3, and day 4 (both with *p* = 0.0068) and on day 2 with significance against day 3 and day 4 (both with *p* = 0.0292) (Figure 12d). Clear differences between the cytokine’s expression levels in the apical and basolateral sides of hBAEC were observed and are represented using scatter plots in Figure S4. According to our results, the polarized secretion of cytokines on the apical compartment may play a key role in modulating the hBAEC response to the viral infections.

## 4. Discussion

This study evaluated the prevalence of SARS-CoV-2 Omicron subvariants, their susceptibility to neutralization by commercial human antibodies, and the mutation profiles within the spike protein region of newly emerged strains. Additionally, we assessed the ability of these subvariants to infect hBAEC, both alone and in combination with common respiratory viruses. The hBAEC were cultured at ALI, allowing them to differentiate into a pseudostratified mucociliary epithelium that closely resembles the in vivo human airway.

Our findings confirmed numerous studies indicating that the spike protein is a highly polymorphic gene containing multiple amino acid substitutions that create a unique pattern to every lineage of Omicron [3,4,5]. When investigating the response of Omicron subvariants in comparison to a parent strain isolated at the beginning of the pandemic against Abs neutralization, we found a weak antiviral activity of AM359b, casirivimab, and bebtelovimab against Omicron JN.1 or its most recent descendants. This effect was similar when evaluating different human sera from individuals infected at the early stages of the pandemic and without receiving vaccination.

Several antibody therapies have been evaluated in COVID-19 patients. The administration of casirivimab/imdevimab (REGEN-COV^®^) reduced the viral load and improved clinical outcomes in hospitalized patients with COVID-19 on low-flow or no supplemental oxygen conditions [30]. This monoclonal antibody combination was authorized for the treatment and post-exposure prophylaxis of patients with COVID-19; however, it showed a more potent neutralization effect against initial SARS-CoV-2 variants compared to the Omicron variant [31]. Indeed, starting January 2024, casirivimab is no longer authorized for therapeutic or post-exposure treatment in COVID-19 patients [32]. Another antibody, bebtelovimab, received emergency-use authorization by the FDA in the US back in 2022, as early therapy in patients with mild-to-moderate COVID-19, especially high-risk adults and children over 12 years old. However, the FDA update from 2024 provided information about reduced activity of bebtelovimab against the Omicron subvariants BQ.1 and BQ.1.1 [33]. This poor response to antibody therapy shown by Omicron is also supported by other studies using early emerging Omicron subvariants [34,35].

The Omicron variant exhibited more than a dozen mutations in the spike protein compared to the original strain isolated in Wuhan at the beginning of the pandemic, which have been linked to changes in viral pathogenesis, transmissibility, and enhanced antibody evasion [36]. Studies have also demonstrated significant reinfection rates and vaccine failure mainly due to the ability of Omicron to escape antibody neutralization [37,38]. In recent years, several studies analyzed the impact of these spike protein mutations and their ability to escape the immune response of the host [39,40]. Another group reported an impaired neutralizing activity of RBD class 3 monoclonal antibodies (mAbs) against XBB and BQ subvariants containing the R346T mutation [41]. In correlation with this and another study [42], we found the R346T amino acid substitution in LB.1, KP.2, and its descendants, KS.1 and XDK, whose response to neutralizing antibodies was also weak, probably explaining their reduced neutralization by sera. In contrast, both KP.3.1.1 and KP.3.1.4 subvariants lack the above-mentioned substitution but harbor the Q493E mutation, which has been previously related to a reduced binding affinity to ACE2 but not a decreased antibody neutralization in a KP.3 strain [43]. This is in alignment with our findings, since the KP.3.1.1 and KP.3.1.4 strains show a better response to neutralization assays within the pool of subvariants that we investigated. In line with this, a recent study suggests that the deletion of a serine in position 31, observed in LB.1, KP.2.3, KP.3, and KP.3.1.1, is the primary cause for reduced neutralizing antibody titers [44], although in our findings LB.1 and KP.3.1.1 did not carry this mutation. It is noteworthy that the dominance of the KP.3.1.1 subvariant was the longest within the prevalent Omicron strains, although its correlation to the mutations on the spike protein has not been proved yet. Broadly expanded within the collection of omicron strains from this study is the polymorphism N969K, previously related to the modified expression levels of the spike protein in the cell surface, which impacts the syncytia formation of the virus and, thus, affects its recognition by the host [45]. Additionally, the amino acid change D796Y present in all the strains from our study except for KP.2 has been correlated to a decreased neutralization by human sera without a significant modulation of the spike protein [46]. All together, these studies help to explain the low responding phenotype to host immunity that these subvariants show in our findings, both against commercial neutralizing antibodies and human sera, highlighting the importance to renovate the composition of vaccines to cover these new circulating subvariants and bring back the immunity levels to the vaccinated population.

The implementation of measures to reduce the transmission of SARS-CoV-2 in the past also helped to reduce the incidence of cases with other respiratory viruses. However, since the beginning of the pandemic, several cases have been reported with SARS-CoV-2 coinfection. With the end of the SARS-CoV-2 pandemic and the lack of these preventive actions, viruses like Influenza and RSV increased their circulation and more cases were reported soon after, providing all the necessary factors for the appearance of coinfections. However, this phenomenon is still poorly investigated, with a prevalence not very well reported and sometimes miss-considered at low frequency [47].

Influenza and RSV are reported as one of the most common respiratory viral illnesses by the CDC, and at higher risk in older adults, young children, people with weakened immune systems, and pregnant women, within other populations [48]. A recent example of coexistence of SARS-CoV-2 and influenza virus was highlighted in a study where authors found the occurrence of three outpatient coinfections in the pediatric population [49]. On the other hand, RSV is the main cause of bronchiolitis worldwide and the most common lower respiratory tract infection, especially in young children [50]. This population is particularly susceptible to respiratory viral coinfections [51], where a high transmission rate of RSV or SARS-CoV-2 has been reported [52]. Immortalized cell lines are commonly used for virus characterization due to their rapid growth, controlled conditions, robustness, and relatively short time requirements. Instead, hAEC provides a more physiologically relevant 3D model for studying viral infections under conditions that closely mimic the human respiratory tract’s environment and cellular functions. These live cells are derived from human donors and can develop stratified epithelium and produce immunological mediators, offering a more accurate representation of the in vivo environment. Several studies have previously investigated the effects of viral monoinfections of seasonal alpha- and betacoronaviruses, SARS-CoV-2, or spike glycoprotein S1 domain from SARS-CoV-2 [13,53,54,55,56], and others evaluated the effects of mAbs using an ALI model [57]. Zarkoob and colleagues investigated the cellular complexity of human alveolar and tracheobronchial ALI tissue models during SARS-CoV-2 WA1/2020 and Influenza A virus, but as monoinfections [58], and another study evaluated the coinfection of SARS-CoV-2 and Influenza coinfection but using an in vitro assay with Calu-3 cell line [59].

Due to the associated worse outcomes that may appear in viral coinfections compared to monoinfections, here we evaluated the SARS-CoV-2 infection of the ancestral WA1/2020 strain and the prevalent Omicron subvariants JN.1 and KP.3.1.1 using hBAEC in an ALI model (Figure 5, Figure 6, Figure 7, Figure 8, Figure 9, Figure 10, Figure 11 and Figure 12). Our results indicated a lower CPE induced by WA1/2020 and lower infection in the apical and basolateral side of the hBAEC compared to the Omicron subvariants, where both JN.1 and KP.3.1.1 showed an increase in the viral load that peaked at 2 days post-infection (Figure 5 and Figure 6). These findings were consistent through several readouts including CPE observations, viral RNA quantification by RT-qPCR, and viral detection by immunofluorescence analysis (Figure 5, Figure 6 and Figure 7) but are in contraposition to previous reports where authors described a less favorable Omicron replication and less severity of infection in the lower respiratory tract [60]. In some cases, this effect has been attributed to viral escape mechanisms against the immunity generated by vaccination and previous infections. Another work published in 2022 reported similar replication between Omicron BA.1 and the Delta variant in human nasal epithelial 3D cultures, but Omicron replication was significantly decreased in lower airway organoids [61]. We believe that the discrepancies with previous studies may be related to differences in the viral cell entry between early circulating strains with the most recent prevalent subvariants, but this needs to be further investigated. In addition, we observed a high production of IFN-β in the inserts infected with WA1/2020, which may be responsible for the low infection with this strain, as a previous study reported that IFN-β treatment effectively block SARS-CoV-2 replication [62]. In addition, the combination of KP.3.1.1 with IFAV_H1N1 or RSV induced more significant damage to the epithelium but did not enhance the apical infection compared to Omicron monoinfection, suggesting that the combination with these two viruses did not attenuate the replication of SARS-CoV-2 in hBAEC.

In summary, hBAEC cultures proved to be a representative model for the characterization of viral respiratory mono- and coinfections in lower airway epithelial cells. Human cells were successfully cultured on permeable support with minimal differentiation requirements, offering valuable insights into the behavior of viruses coexisting in the lung environment, while avoiding the use of animal models. We recognize that additional characterization should include the use of lung organoids, as they allow for the investigation of viral infections’ impact on a broader range of cell types beyond epithelial cells. The data presented here contribute to the understanding of SARS-CoV-2 evolution and infection behaviors when affecting the human lung epithelial cells as a monoinfection or in combination with other respiratory viruses, as well as suggesting that there are still knowledge gaps related to SARS-CoV-2 infection.

## Data Availability

The original contributions from this study are included in this article. Further inquiries can be directed at the corresponding author.

## Supplementary Materials

The following supporting information is provided in an additional file: https://www.mdpi.com/article/10.3390/v17070918/s1, Figure S1. SARS-CoV-2 Virus Stocks Characterization; Figure S2. Influenza H1N1 and RSV Virus Stocks Characterization; Table S1. SARS-CoV-2 virus strains; Table S2. Sequence of primers (5′-3′) and probes used in this study; Figure S3. Virus recovery from viral infection of hBAEC in the ALI model; Figure S4. Scatter plot depicting the maximum fold difference of cytokine expression level in hBAEC, between the apical and basolateral sides.

## Abbreviations

The following abbreviations are used in this manuscript and are listed here in alphabetical order:

A/A	antibiotic/antimycotic
AF	Alexa Fluor
ALI	air-liquid interface
ANOVA	Analysis of variances
Abs	antibodies
BEI Resources	Biodefense and Emerging Infections Research Resources
BF	brightfield
BSA	bovine serum albumin
CDC	Centers for Disease Control
CDI	Center for Discovery and Innovation
COVID-19	Coronavirus Infectious Disease 2019
CPE	cytopathic effect
CV	crystal violet
DAPI	4′,6-diamidino-2-phenylindole
DMEM	Dulbecco’s Modified Eagle Medium
EMEM	Eagle’s Minimum Essential Medium
FBS	fetal bovine serum
gDNA	genomic DNA
HA	haemagglutinin
hAEC	human airway epithelial cells
hBAEC	human airway epithelial cells derived from bronchiolar tissue
HCoP	human convalescent plasma
Hep-2	Human cervix epithelial cells
HMH-BioR	Hackensack Meridian Health BioRepository
IFAV_H1N1	Influenza A virus
LOQ	limit of quantification
M	matrix
mAbs	monoclonal antibodies
MCC	Mander’s colocalization coefficient
MDCK	Madin-Darby Canine Kidney
MOI	Multiplicity of infection
NA	neuraminidase
NBF	neutral buffered formalin
NJDOH	New Jersey Department of Health
NTD	N-terminal domain
NT_50_	Neutralization titers at 50%
ORF2	open reading frame 2
PCC	Pearson’s correlation coefficient
PET	polyester
PFU	Plaque forming unit assay
RBD	receptor-binding domain
RSV	Respiratory Syncytial Virus
RT	room temperature
SARS-CoV-2	severe acute respiratory syndrome coronavirus 2
SD	standard deviation
SE	standard error
TPCK	TPCK-treated trypsin from bovine pancreas
WA1/2020	Washington strain
WHO	World Health Organization
