# Supplementary material for: Genetic and Immunological Profiling of Recent SARS-CoV-2 Omicron Subvariants: Insights into Immune Evasion and Infectivity in Monoinfections and Coinfections
Source: Viruses. 2025 Jun 27;17(7):918. doi: 10.3390/v17070918 (PMC12300583; doi:10.3390/v17070918)
Supplement: Supplementary file 1 [file viruses-17-00918-s001.zip › viruses-3699250-supplementary.pdf]

SUPPLEMENTARY INFORMATION FOR PUBLICATION

Genetic and Immunological Profiling of Recent SARS-CoV-2 Omicron Subvariants: Insights into Immune Evasion and Infectivity in Monoinfections and Coinfections

Nadine Alvarez *, Irene Gonzalez-Jimenez, Risha Rasheed, Kira Goldgirsh, Steven Park and David S. Perlin *

Center for Discovery and Innovation, Hackensack Meridian Health, 111 Ideation Way, Nutley, NJ 07110, USA; irene.gojim@gmail.com (I.G.-J.); risha.rasheed@hmh-cdi.org (R.R.); kira.goldgirsh@hmh-cdi.org (K.G.); steven.park@hmh-cdi.org (S.P.)
* Correspondence: nadine.alvarez@hmh-cdi.org (N.A.); david.perlin@hmh-cdi.org (D.S.P.)

<u>Content</u>

- **Figure S1.** SARS-CoV-2 Virus Stocks Characterization.
- **Figure S2.** Influenza H1N1 and RSV Virus Stocks Characterization.
- **Table S1.** SARS-CoV-2 virus strains.
- **Table S2.** Sequence of primers (5′-3′) and probes used in this study.
- **Figure S3.** Virus recovery from viral infection of hBAEC in the ALI model.
- **Figure S4.** Scatter plot depicting the maximum fold difference of cytokine expression level in hBAEC, between the apical and basolateral sides.

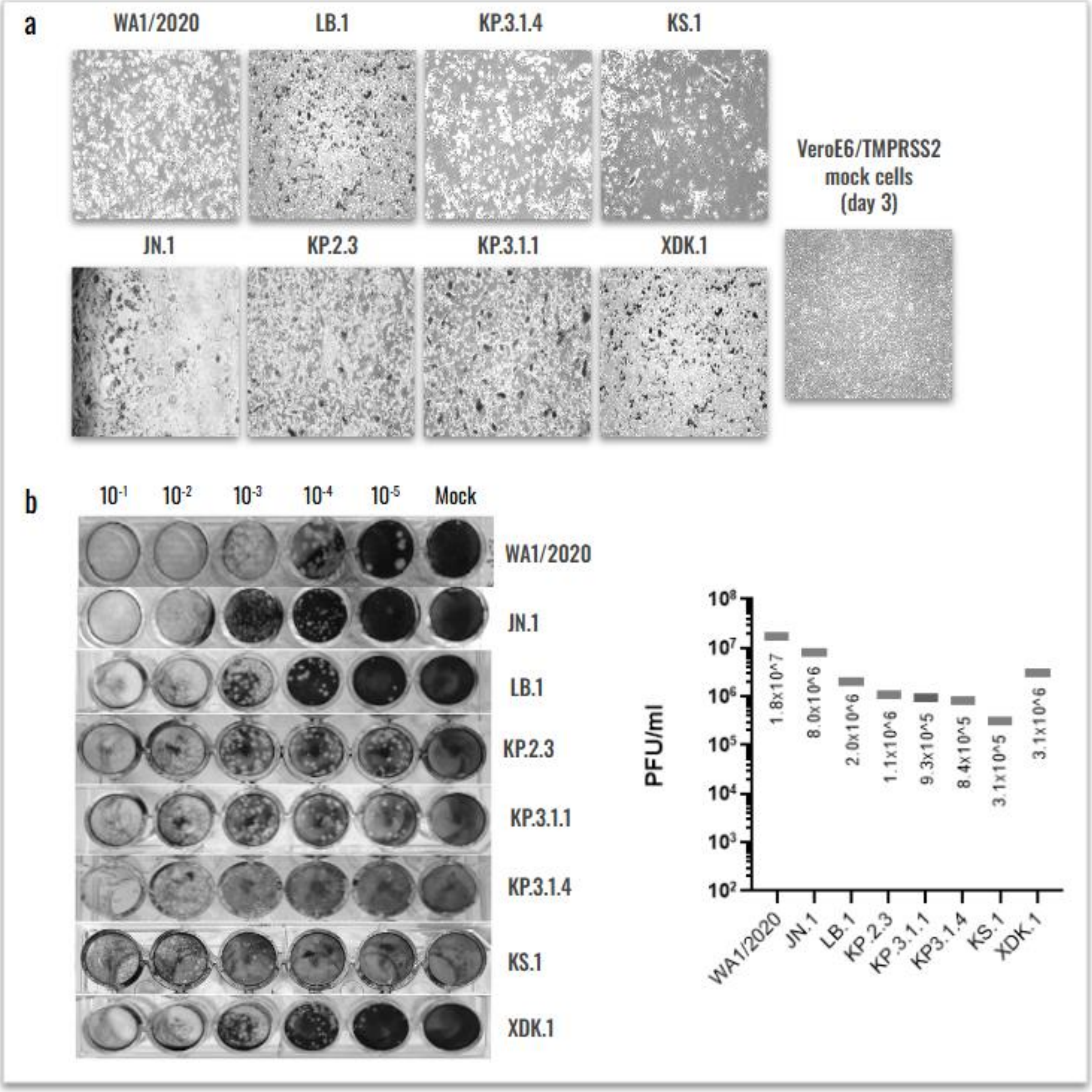

Figure S1. SARS-CoV-2 Virus Stocks Characterization. (a) Cytopathic effect (CPE) of different SARS-CoV-2 strains in VeroE6/TMPRSS2 cells at 3 days post-infection in T175 flasks. (b) VeroE6/TMPRSS2 cells were seeded in 24-well plates at a density of 2 x 10^5 cells per well, and viral plaques were visualized on day 3 post-infection after staining with 0.35% crystal violet. The numbers at the top represent the viral inoculum dilution (10^{-1} to 10^{-5}). Mock-infected cells were included as controls in each assay. Virus titer is expressed in PFU/ml.

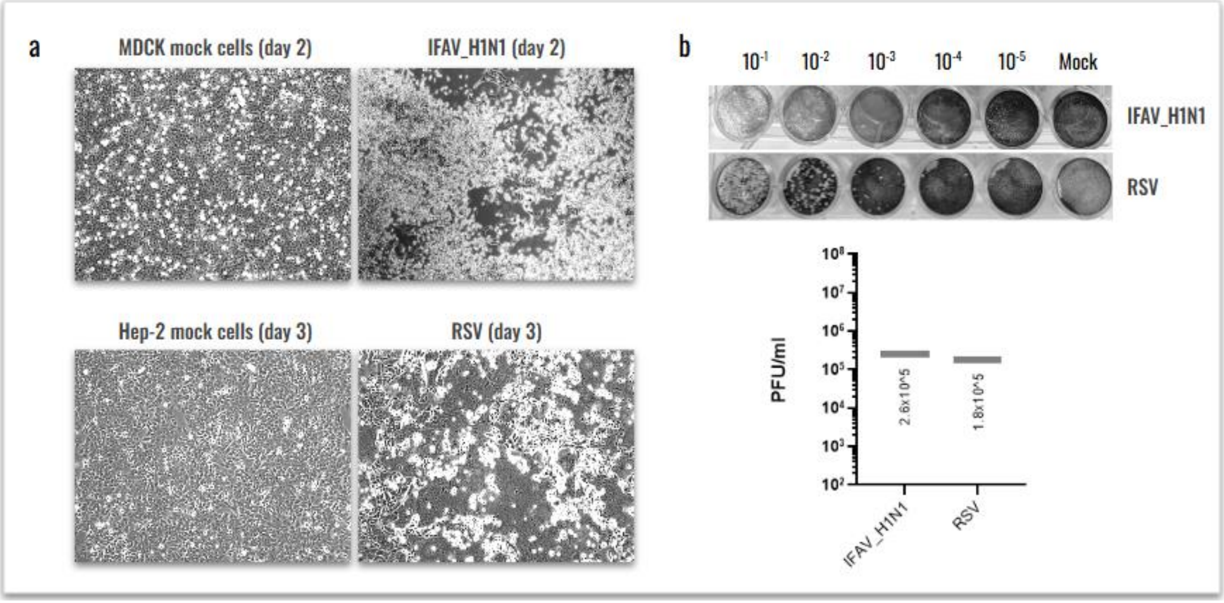

Figure S2. Influenza H1N1 and RSV Virus Stocks Characterization. (a) Cytopathic effect (CPE) of IFAV_H1N1 in MDCK cells and RSV in Hep-2 cells using T175 flasks, observed at 2- and 3-days post-infection, respectively. (b) MDCK and Hep-2 cells were seeded in 24-well plates at a density of 2 x 10^5 cells per well. IFAV_H1N1 viral plaques visualized after staining with 0.35% crystal violet, and RSV viral plaques visualized after staining with 0.05% neutral red, at 4- and 6-days post-infection, respectively. The numbers at the top represent the viral inoculum dilution (10^{-1} to 10^{-5}). Mock-infected cells were included as controls in each assay. Virus titer is expressed in PFU/ml.

Table S1. SARS-CoV-2 virus strains

BEI Resources	Cat. No.	Lot. No.
USA-WA1/2020	NR-53873	70039812
Omicron B.1.1.529	NR-56461	70049434
Omicron BA.2	NR-56520	70051592
Omicron BA.2.75	NR-58700	70055732
Omicron BA.5	NR-58616	70053501
Omicron BA.5	NR-56798	70053884
Omicron BQ.1	NR-58975	70057300
Omicron BF.5	NR-58716	70055929
HMH-Biorepository	Code	GISAID
Delta	CVD198	N/A
Omicron BA.5.5	CVD299	EPI_ISL_14000783
Omicron XBB.1.5	CVD357	EPI_ISL_16346196
Omicron XBB.1.9.2	CVD374	EPI_ISL_17588025
Omicron XBB.1.16.1	CVD372	EPI_ISL_17617817
Omicron EG.5.1	CVD385	EPI_ISL_18107634
Omicron BA.2.86.1.1 (JN.1)	CVD408	EPI_ISL_18669842
Omicron LB.1	NS5	N/A
Omicron LB.1.7	NS54	EPI_ISL_19386980
Omicron KP.2.3	NS73	EPI_ISL_19386982
Omicron KP.3.1.1	NS75	EPI_ISL_19386979
Omicron KP.3.1.4	NS51	EPI_ISL_19350851
Omicron KS.1	NS56	EPI_ISL_19287340
Omicron XDK.1	NS3	EPI_ISL_19350855

Table S2. Sequence of primers (5′-3′) and probes used in this study.

Spike protein target-based sequencing		
Spike protein	For	5′-GGTATATGCGCTAGTTATCAG-3′
	Rev	5′-CAAATTTGCAGCAGGATCCAC-3′
RT-qPCR		
SARS-CoV-2 (E_Sarbeco)	For	ACAGGTACGTTAATAGTTAATAGCGT
	Rev	ATATTGCAGCAGTACGCACACA
	Probe	FAM-ACACTAGCCATCCTTACTGCGCTTCG-BHQ-1
IFAV_H1N1 (HA)	For	GGGAGAATGAACTATTACTGG
	Rev	AGTAGAAACAAGGGTGTTTTT
IFAV_H1N1 (NA)	For	AGCAAAAGCAGGAGTTTAAAATG
	Rev	CCTATCCAAACACCATTGCCGTAT
IFAV_H1N1 (Matrix)	For	GACCRATCCTGTCACCTCTGAC
	Rev	AGGGCATTYTGGACAAAKCGTCTA
	Probe	FAM-TGCAGTCCTCGCTCACTGGGCAGC- BHQ-1
RSV (F protein)	For	AGGTCCTGCACCTAGAAGGGGA
	Rev	TGCTGCAGCTTTGCTTGTTCACA
	Probe	(FAM) AGCTGACTACAGCCTTGTTTGTGGA (TAMRA)
RSV (N protein)	For	GCTCTTAGCAAAGTCAAGTTGAATGA
	Rev	TGCTCCGTTGGATGGTGTATT
	Probe	(FAM)ACACTCAACAAAGATCAACTTCTGTCATCCAGC (TAMRA)
Cytokine expression		
IL6	For	CACACAGACAGCCACTCACC
	Rev	CCTCAAACTCCAAAAGACCA
CCL5	For	CGCTGTCATCCTCATTGCTA
	Rev	ACACACTTGGCGGTTCTTTC
TNFα	For	CTCCTCACCCACACCATCA
	Rev	GGAAGACCCCTCCCAGATAG
IFN-β	For	ATTGCCTCAAGGACAGGATG
	Rev	GCTGCAGCTGCTTAATCTCC
IFN-λ1	For	GGACGCCTTGGAAGAGTCACT
	Rev	AGAAGCCTCAGGTCCCAATTC
CXCL10	For	TCCACGTGTTGAGATCATTGC
	Rev	TCTTGATGGCCTTCGATTCTG
IL10	For	GCTGGAGGACTTTAAGGGTTACCT
	Rev	CTTGATGTCTGGGTCTTGGTTCT
IL13	For	ACAGCCCTCAGGGAGCTCAT
	Rev	TCAGGTTGATGCTCCATACCAT
IRF3	For	TCGTGATGGTCAAGGTTGT
	Rev	AGGTCCACAGTATTCTCCAG
IRF7	For	GCAAGTGCAAGGTGTACTG

	Rev	CACCAGCTCTTGGAAGAAGA
B-actin	For	TACGCCAACACAGTGCTGTCT
	Rev	TCTGCATCCTGTCGGCAAT

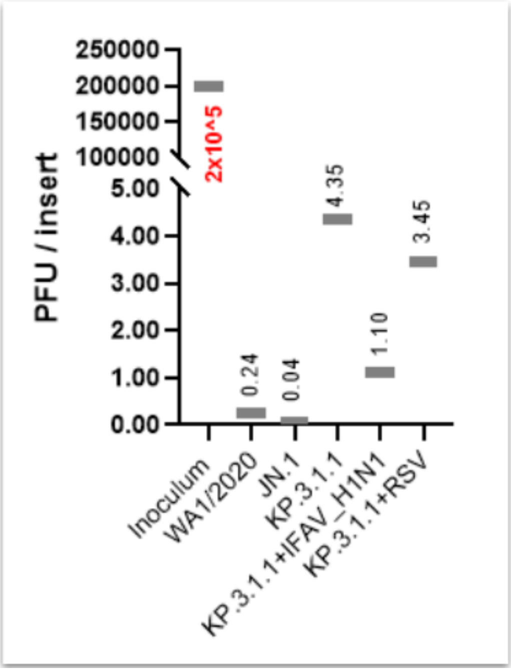

Figure S3. Virus recovery from viral infection of hBAEC in the ALI model. The virus inoculum used for the infection of hBAEC with each condition (mono- or coinfection) was collected after allowing 1 h of infection at 37°C with 5% CO_2. The recovered samples were analyzed by PFU assay in VeroE6/TMPRSS2 cells using 24-well plates. Viral plaques were visualized and counted at 3 days post-infection after staining with 0.35% crystal violet, and virus titer was calculated and is represented here as PFU/ml. As a reference, the titer for each virus inoculum is included in the graph and highlighted in red (2×10^5 PFU / insert).

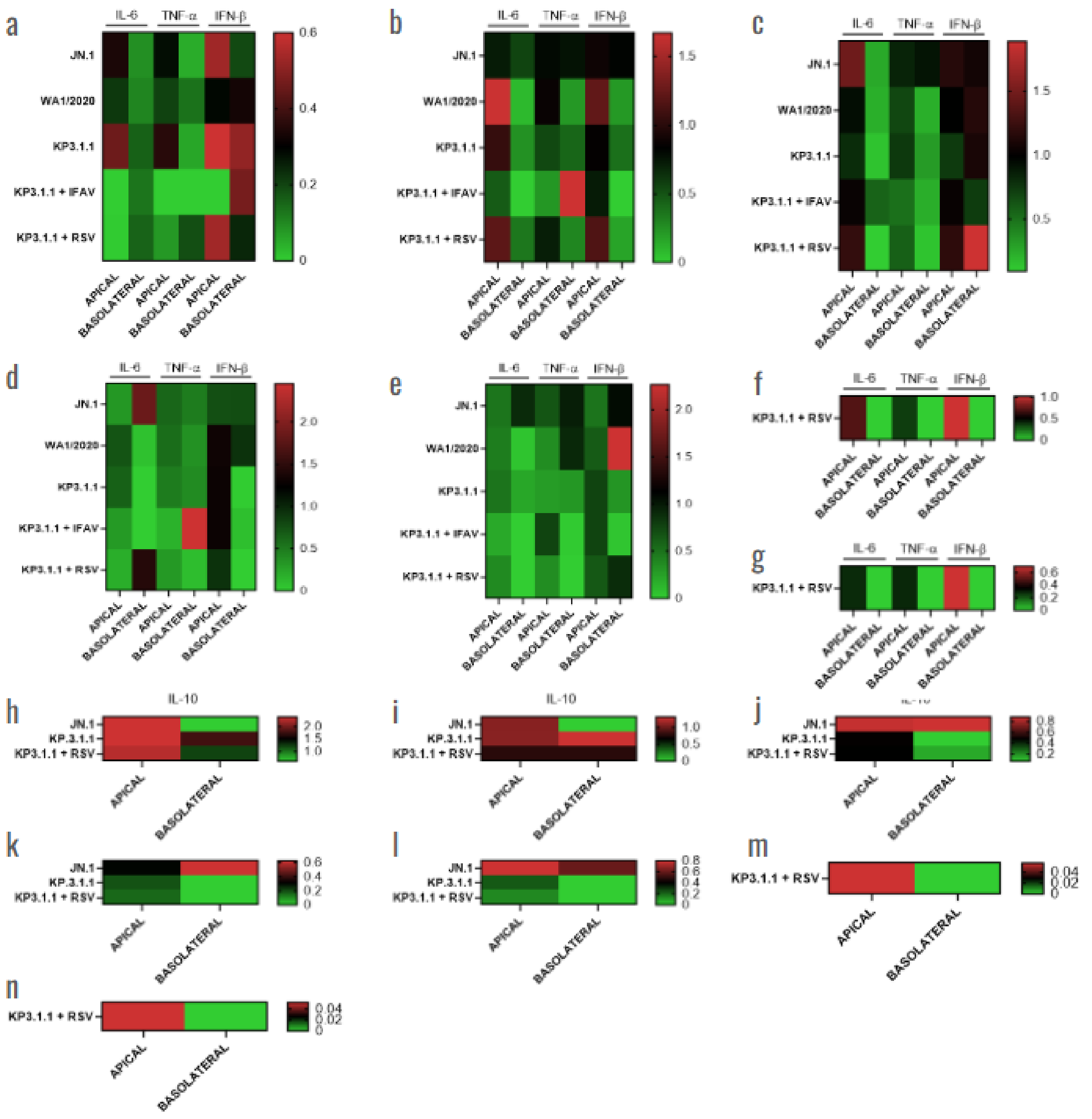

Figure S4. Scatter plot depicting the maximum fold difference of cytokine expression level in hBAEC, between the apical and basolateral sides. IL-6, TNF-**α** and IFN-**β** levels are represented for all infections in graphs a) day 0; b) day 1; c) day 2; d) day 3; e) day 4; f) day 5; g) day 6. IL-10 levels are represented for JN.1, KP.3.1.1 and KP.3.1.1+RSV in graphs h) day 0; i) day 1; j) day 2; k) day 3; l) day 4; m) day 5; n) day 6.
